# The role of Treg subtypes in glomerulonephritis

**DOI:** 10.1007/s00441-020-03359-7

**Published:** 2020-12-14

**Authors:** G. R. Herrnstadt, O. M. Steinmetz

**Affiliations:** grid.13648.38 0000 0001 2180 3484 III. Department of Medicine, University Medical Center Hamburg-Eppendorf, Martinistrasse 52., 20246 Hamburg, Germany

## Abstract

While Th1 and Th17 T effector cells are pathogenic drivers of glomerulonephritis (GN), regulatory T cells (Tregs) potently protect from renal tissue injury. Recently, it has become evident that different Treg subtypes exist. Among these are lineage specific Treg1 and Treg17 cells, which are specialized to down regulate either Th1 or Th17 T effector cell responses. Interestingly, programming of specialized Tregs and the corresponding T helper effector cells depend on the same lineage specific master transcription factors Tbet (Th1/Treg1) and STAT3 (Th17/Treg17). Furthermore, early control of T effector cell priming in secondary lymphoid organs by specialized Tregs was described. One central mechanism of T effector cell control by the corresponding Treg subtype seems to be expression of the same chemokine receptor repertoire, which facilitates their co-localization. More recently, another intriguing Treg subset was identified, which expresses Foxp3 together with the Th17 characteristic transcription factor RORγt. While these Foxp3^+^RORγt^+^ Tregs were shown to be highly immunosuppressive, studies in GN also identified pro-inflammatory potential via secretion of IL-17. Many questions regarding this unusual Treg subset remain, including their origin, stability, and mechanisms of action. Further characterization of the renal Treg landscape during GN will help to identify novel immunosuppressive mechanisms and develop successful Treg-directed therapies. In this review, we summarize the currently available data about specialized Treg subsets and discuss their role in GN.

## Introduction

CD4^+^ T effector cells (Teff) and in particular the T helper 1 (Th1) and 17 (Th17) lineages are crucial pathogenic mediators of crescentic glomerulonephritis (GN) (Krebs et al. 2017; Kurts et al. 2013; Phoon et al. 2008; Steinmetz et al. 2011). Interestingly, Th17 cells, characterized by activation of the master transcription factor RORγt and the production of IL-17A+F, showed high abundancy in nephritic kidneys of patients with ANCA-associated vasculitis and significantly aggravated crescentic nephritis in experimental models (Krebs et al. 2016; Paust et al. 2009; Steinmetz et al. 2011). This indicates their central pathogenic importance and highlights their therapeutic potential. Regulatory T cells (Treg) in contrast have the capacity to counter-regulate over-shooting immune responses, which has been shown to protect from a large number of autoimmune diseases including GN (Kurts et al. 2013; Steinmetz et al. 2010; Vignali et al. 2008). As a consequence, Tregs became promising therapeutic targets (Bluestone and Tang 2018; Raffin et al. 2019). Human studies of ANCA-associated vasculitis, Systemic Lupus Erythematosus, Goodpasture syndrome, and IgA nephropathy revealed dysregulation of Treg homeostasis and function, further fueling the interest in Treg based therapies for these diseases (Abdulahad et al. 2007; Free et al. 2013; Ooi et al. 2017; Von Spee-Mayer et al. 2016; Yang et al. 2015).

Several years back, identification of Tregs was based on observations, that T cells with high expression of the IL2 receptor alpha chain (CD25) had suppressive function in vitro and in vivo (Baecher-Allan et al. 2001; Dieckmann et al. 2001; Jonuleit et al. 2001; Levings et al. 2001; Ng et al. 2001; Sakaguchi et al. 1995; Taams et al. 2002). By that time, an important study from Wolf et al*.* could for the first time show a strong protective effect of CD25^hi^ T cells in an experimental model of acute glomerulonephritis using adoptive T cell transfers (Wolf et al. 2005). Renal tissue protection after transfer of CD25^hi^ T cells into nephritic recipients was accompanied by a marked decrease of renal CD4^+^ T cells, CD8^+^ T cells, and macrophages. Additionally, CD25^hi^ T cells effectively suppressed Th1 responses, systemically and locally in the kidneys. The later discovery of Foxp3 as the specific master transcription factor of Tregs in both, humans and rodents, marked a further milestone in Treg biology (Fontenot et al. 2017; Hori et al. 2017; Khattri et al. 2017). Two studies analyzed effects of Foxp3^+^ Treg depletion in the nephrotoxic nephritis model (NTN) of acute GN, using injection of diphtheria toxin (DT) into mice with genetic introduction of the DT receptor under control of the Foxp3 gene promoter (DEREG). Both studies reported striking aggravation of experimental glomerulonephritis, in a predominantly Th1-mediated fashion (Ooi et al. 2011; Paust et al. 2011). Interestingly, these studies also revealed, that Tregs derived from immunized donors showed higher ex vivo suppressive capacity, compared with Tregs from naïve donors. This suggested that immune stimuli enhance Treg function or that in part antigen specificity might play a role (Ooi et al. 2011). Also, using immunohistochemical staining, as well as reporter mice with Foxp3 auto-fluorescence, both studies for the first time identified Tregs inside nephritic renal tissue. This pointed towards infiltration of Tregs into target organs to mediate their function, rather than their sole localization in secondary lymphatic organs. A few years later, another important tool for Treg studies became available. Genomic introduction of cre recombinase under control of the Foxp3 promotor allowed to generate mice with Treg selective deficiency for defined key molecules (Rubtsov et al. 2008). Ostmann et al. used this approach and identified IL-10 as a key cytokine used by Tregs to protect from experimental crescentic GN (Ostmann et al. 2013). A further key method for Treg studies was the development of Foxp3 fluorescence reporter mice. These opened up the opportunity for adoptive transfer studies of Foxp3^+^, rather than CD25^+^ T cells, allowing to more accurately identify Tregs and better characterize their function in GN. Using this technology, Kluger et al. could demonstrate that injection of Foxp3^+^ Tregs into mice with acute GN significantly ameliorated the course of nephritis (Kluger et al. 2016a). While all above mentioned studies supported an anti-inflammatory role of Tregs in GN, it remained unclear, whether antigen specificity played a role. In this respect, a recent comprehensive study by Ooi et al. showed that generation of Tregs is crucially influenced by the way nephritogenic antigens are presented in the MHC class II complex. Furthermore, and importantly, Tregs with specificity against the Goodpasture antigen conferred protection from disease (Ooi et al. 2017).

In conclusion, a large number of studies, analyzing Treg function in GN, demonstrated strong anti-inflammatory and reno-protective effects. However, several open questions remain. In light of the discovery of a growing number of functionally different T effector cell lineages, it seems unlikely that one master Treg population with an omni-potent immunosuppressive phenotype exists, which can equally well control each different T effector cell population. It became thus likely that Tregs do not resemble a homogenous population. Rather, in analogy to their pathogenic pro-inflammatory T effector cell counterparts, different sub-specialized Treg population might exist. The following paragraphs of this review will focus on this aspect and summarize currently available data on Treg subtypes in GN.

## Treg17 cells: regulators of Th17 immunity

Landmark studies regarding T helper cell biology revealed unique transcriptional programs to define Th1, Th2, or Th17 lineage commitment. This allowed discrimination between T helper effector cell subtypes, based on expression of their unique and distinguishing master transcription factors (Ivanov et al. 2006; Manel et al. 2008; Szabo et al. 2000, 2002; Zheng and Flavell 1997). Following this observation, the idea of lineage-specific master transcription factors, which might also define particular and functionally different Treg subtypes, was born. Th17 commitment of naïve T cells is largely driven by STAT3 activation (Ivanov et al. 2006; Yang et al. 2008; Zhou et al. 2007). Consequently, the role of STAT3 for Treg function was investigated. Indeed, first studies with constitutional Treg-restricted deletion of STAT3 led to overshooting Th17 responses, accompanied by spontaneous colitis development in mice. Th1 and Th2 responses, on the other hand, remained unaltered. In search of the underlying mechanisms, the authors found that expression of the chemokine receptor CCR6 on Tregs depended on STAT3 activation. Since Th17 cells also highly express the CCR6, the authors postulated that STAT3 positive Tregs might regulate Th17 responses by close co-localization via use of the CCR6 (Chaudhry et al. 2009). Since IL-17 and Th17 cells play well established pathogenic key roles in crescentic GN (Gan et al. 2010; Krebs et al. 2017; Ooi et al. 2009; Paust et al. 2009; Steinmetz et al. 2011; Summers et al. 2009, 2014), our group decided to study this aspect further. In line with the previous findings, cell type–restricted ablation of STAT3 in Foxp3^+^ Tregs led to significant aggravation of renal injury in the NTN model of GN, with skewing of renal and systemic immune responses towards Th17 immunity. We thus proposed the term “Treg17” cells for STAT3-dependent Tregs, in analogy to their pro-inflammatory Th17 counter parts. Mechanistically, we could confirm that STAT3 deficiency indeed resulted in a profound reduction of CCR6 expression on Tregs. This resulted in substantial impairment of Treg infiltration into inflamed kidneys, while systemic Treg frequencies remained unaltered. Remarkably, STAT3 deletion had no effects on Treg survival, activation, or suppressive capacity. These findings suggested that directional migration into target organs, specifically into areas of Th17 cell infiltration via use of the CCR6, is the major mechanism of Th17 control used by Treg17 cells. This hypothesis was supported by human data, showing that CCR6^+^ Tregs closely co-localized with CCR6^+^ Th17 cells in kidneys of patients with ANCA-associated GN, which extends the concept of Treg17 cells to the human immune system. Additionally, Tregs from patients with Hyper-IgE Syndrome (HIES), a primary immunodeficiency caused by dominant negative STAT3 mutations, showed significantly reduced surface expression of CCR6. Percentages of systemic Tregs in the blood of HIES patients, in contrast, were unaltered compared with healthy controls. This further supported a trafficking, rather than a developmental or homeostasis problem (Kluger et al. 2014).

In a follow-up study, our group then investigated the role of Treg17 cells during the course of chronically developing lupus nephritis using the pristane induced model. Similar to our findings from acute crescentic GN, deletion of STAT3 in Tregs resulted in aggravation of peritonitis, pulmonary vasculitis, and lupus nephritis. Analysis of immune responses in all affected organs uniformly revealed selectively overshooting Th17 responses, with no alterations of Th1 or Th2 immunity. As underlying mechanism, we again found reduced CCR6 expression on Tregs, impairing their trafficking into areas of Th17 inflammation (Kluger et al. 2016b). However, whether in addition to optimizing their trafficking properties, STAT3 activation also regulates key suppressor molecules of Treg17 cells, which contribute to their lineage specific functions, needs to be further evaluated.

**Fig. 1:**
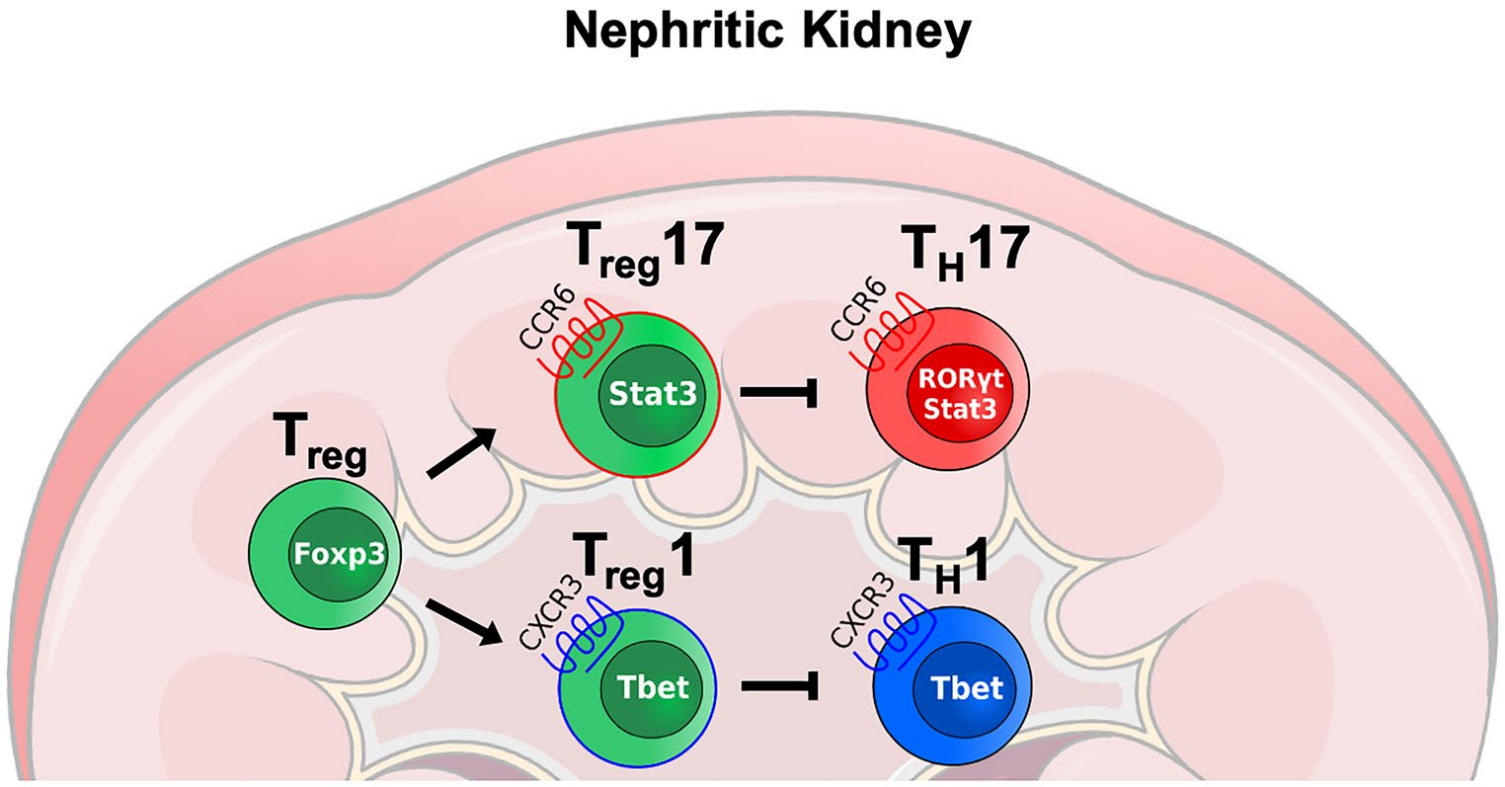
In analogy to T helper cell differentiation, lineage specific transcription factors can be activated in naive Foxp3^+^ Tregs under inflammatory conditions. Tbet activation in Tregs induces a Treg1 phenotype, characterized by expression of chemokine receptor CXCR3, which optimizes them for co-localization and control of CXCR3^+^ Th1 cells. Likewise activation of STAT3 results in generation of CCR6^+^ Treg17 cells, which are tailor made for suppression of Th17 immunity in glomerulonephritis via trafficking into the same areas as CCR6^+^ Th17 cells

In summary, STAT3 activation in Tregs induces a CCR6^+^ Treg17 phenotype in mice and humans, which is uniquely equipped to suppress pathogenic Th17 responses during the course of acute and chronic glomerulonephritis (Fig. 1).

## Treg1 cells: regulation of type 1 responses and beyond

Shortly after the discovery of STAT3-dependent Treg17 cells had suggested the concept of T effector cell lineage-specific Treg subtypes, another important study unraveled Treg-mediated mechanisms of Th1 counter regulation. In this regard the role of the Th1 lineage master transcription factor Tbet for Treg biology was investigated. In analogy to STAT3, which is shared between Th17 and Treg17 cells, Koch et al. could show that activation of the Th1 master transcription factor Tbet occurs under Th1-type inflammatory conditions and induces a “Treg1” phenotype in peripheral Tregs. Importantly, this equipped them to specifically suppress Th1 responses (Koch et al. 2009). Mechanistically, Tbet promoted Treg expression of the chemokine receptor CXCR3, which is also crucial for trafficking of pathogenic Th1 cells to sites of inflammation. Shared expression of CXCR3 on Th1 and Treg1 cells thus facilitates close co-localization of both cell types. This concept was proven by a subsequent study in acute GN. Using the NTN model, Paust et al. could show that Treg-specific deletion of CXCR3 led to a significant reduction of Treg frequencies in nephritic kidneys. Importantly, this caused an overwhelming renal Th1 response with aggravation of histological and functional injury. Furthermore, CXCR3 staining of renal biopsies from patients with ANCA vasculitis confirmed the existence of Treg1 cells in human GN and—in line with the murine findings—showed close co-localization of CXCR3^+^ Tregs with CXCR3^+^ T effector cells (Paust et al. 2016). Since it remained unclear, whether indeed Tbet was responsible for CXCR3 expression in Tregs, our group addressed this specific aspect. Time course analyses showed that Tbet^+^ Treg1 cells expressed CXCR3 and expanded in parallel to Tbet^+^ Th1 cells in nephritic kidneys during NTN, which suggested some kind of cross regulation. Indeed, Treg-restricted inactivation of Tbet resulted in complete absence of CXCR3 on Tregs. As a result, naïve mice lacking Tbet in Tregs developed spontaneously overshooting Th1 responses. This hyper Th1 phenotype was even aggravated after induction of crescentic GN (NTN) and resulted in worsening of renal outcome. Mechanistically, our studies showed reduced capacity of Tbet-deficient Tregs to traffic into inflamed kidneys, which equaled the defects observed in mice with selective CXCR3 deficiency on Tregs. In vitro and in vivo suppressive capacity of Tbet inactivated Tregs, in contrast, remained unaltered (Nosko et al. 2016; Yu et al. 2015). This finding even more highlighted the importance of CXCR3 expression on Treg1 cells for their capacity to suppress Th1 responses. It must, however be noted that one study analyzing the function of Tbet^+^ Tregs found contrasting results. While the authors could confirm Tbet dependency of CXCR3 expression on Tregs, they did not find overshooting Th1 responses nor aggravation of disease in models of encephalitis or colitis in the absence of Tbet^+^ Tregs (McPherson et al. 2015). These observations still remain unexplained and indicate that the requirement of Treg1 cells for control of pathogenic Th1 responses might be organ and context dependent. Furthermore, a study by Xiong et al. surprisingly reported that expression of CXCR3 on Tbet^+^ Tregs was not only required for trafficking into target organs but also for afferent lymphatic migration to draining lymph nodes, in order to suppress systemic T cell activation (Xiong et al. 2016).

In addition, competitive co-transfer studies of wild type and Tbet-deficient Tregs revealed yet another Tbet-mediated function, different from Th1 regulation and Treg trafficking. Two independent studies, including in GN, found that in addition to induction of CXCR3 expression, Tbet is also required for optimal Treg homeostasis and fitness (Koch et al. 2009; Nosko et al. 2016). This indicated that the effects of Tbet expression in Tregs extend beyond the mere optimization for control of Th1 immunity. A more recent follow-up study using novel state of the art transgenic mouse lineages fully confirmed and extended this and the other above described observations (Levine et al. 2017). In addition, intricate and comprehensive experiments performed by the authors revealed previously unrecognized requirement of Tbet^+^ Tregs for control of infection-induced CD8 immunity. Conversely, however, a further important study reported another unexpected function of Tbet expressing Tregs regarding the CD8 compartment. The authors found that Tbet^+^ Tregs promote TGFß dependent generation, rather than downregulation, of tissue resident memory CD8^+^ T cells (Trm) in an infection model. As a consequence, the absence of Tbet^+^ Tregs resulted in lower Trm frequencies, leading to increased pathogen burden (Ferreira et al. 2020). Whether Tbet^+^ Tregs also shape CD8 immunity and/or renal Trm cell development during crescentic GN and whether this affects disease outcome is currently not known and will need to be investigated in future studies. Taken together, Tbet expression in Tregs induces a Treg1 type transcriptional program, necessary for optimizing control of pathogenic Th1 immune responses in various autoimmune pathologies, including crescentic GN. The mechanistic basis seems to be shared expression of chemokine receptor CXCR3 on both Tbet^+^ Th1 and Treg1 cells (Fig. 1). In addition, Tbet activation enhances Treg general fitness, necessary for the control of GN.

Apart from these two key functions, Tbet was shown to facilitate Treg egress from inflamed tissues, to facilitate migration into draining lymph nodes for the control of antigen specific T cell activation. Finally, Tbet seems to equip Tregs with both, the potential to downregulate CD8 immunity but also to induce Trm cells with as of yet unknown consequences for GN.

## CCR7^+^ Tregs: guardians of the secondary lymphatic organs

**Fig. 2:**
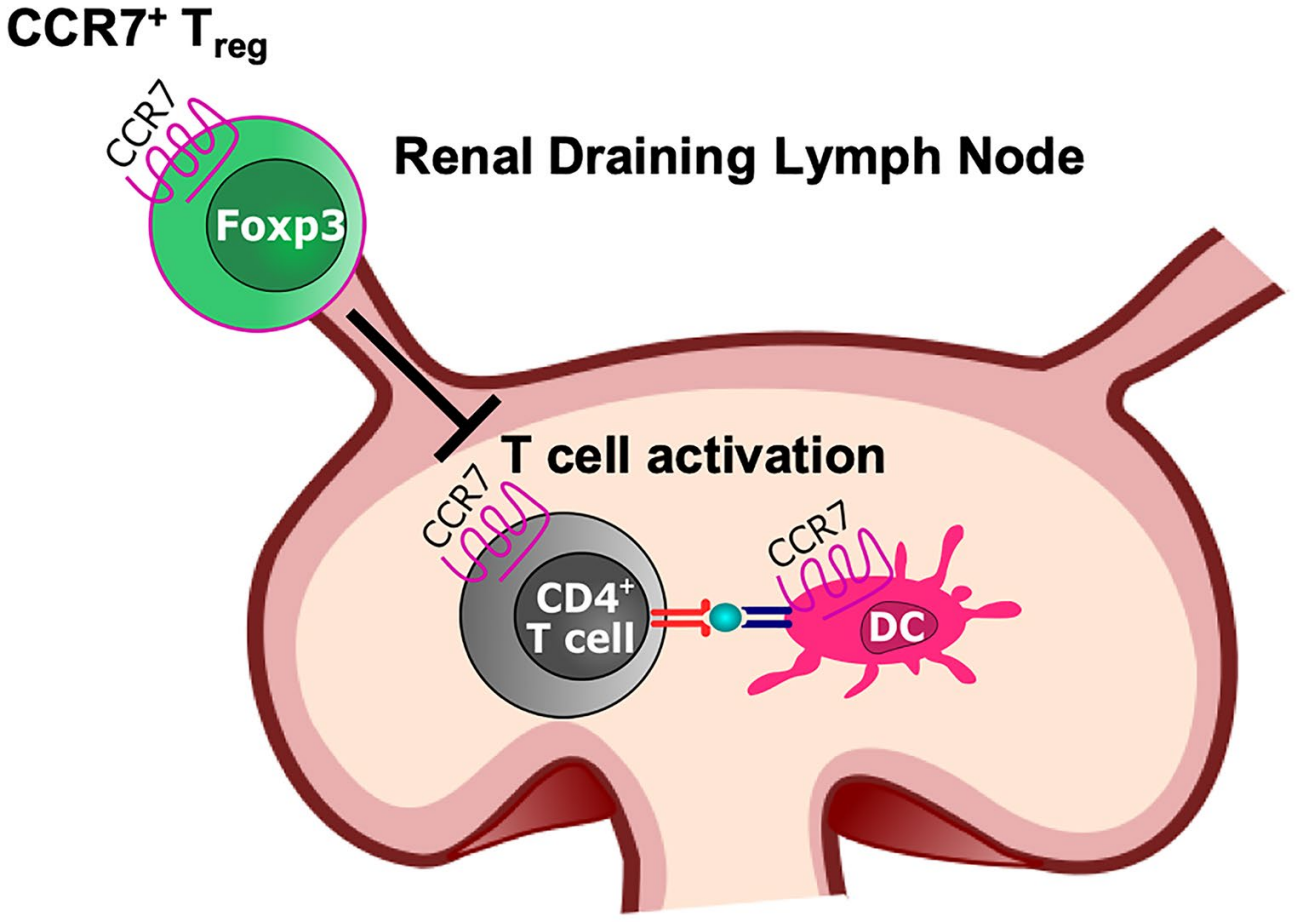
CCR7 expression empowers Tregs to migrate into secondary lymphatic organs, such as the renal lymph node, to suppress activation of naïve CCR7^+^ T cells by antigen-presenting CCR7^+^ dendritic cells (DCs)

As explained above, Tbet^+^ Treg1 cells and STAT3-dependent Treg17 cells control their respective T helper cell counter parts largely via chemokine receptor mediated trafficking to sites of inflammation, as, e.g., the renal interstitium during GN. Initiation of immune responses, however, takes place in secondary lymphoid organs, like spleen and lymph nodes. It is thus tempting to speculate that specialized Tregs might also participate in control of this process. In this respect, the chemokine receptor CCR7 has been identified as crucial mediator. Keynote studies have identified an essential role of CCR7 expression for the egress of antigen-bearing dendritic cells from peripheral sites of inflammation into secondary lymphatic organs. In parallel, naïve T helper cells also show homeostatic CCR7 expression and thus co-localize with antigen laden CCR7^+^ DCs, which is essential for initiation of antigen specific T cell responses (Förster et al. 2008). Interestingly, earlier studies revealed that not only effector T cells but also Tregs routinely use the CCR7 to migrate to sites of antigen-specific T cell activation (Schneider et al. 2007). CCR7^+^ Tregs were thus identified as a specialized Treg subset, which becomes active early on during development of immune responses and guards the initiation process. Regarding the field of nephrology, a keynote study by Eller et al*.* demonstrated high functional relevance of this process for acute GN. The authors could show that pan-CCR7 deficiency resulted in aggravation of acute experimental glomerulonephritis. Analysis of the underlying mechanisms revealed a marked reduction of Treg numbers in renal lymph nodes and spleens. Furthermore, the authors could show that lack of CCR7 resulted in higher T effector cell activation, as well as higher antibody titers against the nephritogenic antigen. Importantly, aggravation of GN, as well as the uncontrolled antibody responses could be reversed by adoptive transfer of CCR7 competent but not CCR7-deficient Tregs. Taken together, these data support the relevance of CCR7^+^ Tregs for GN, most likely via control of T cell priming at systemic sites of immune activation (Eller et al. 2010; Steinmetz et al. 2010). Thus, besides onsite regulation of nephritogenic effector cells in the inflamed kidneys, specialized Treg subtypes might also play important roles in secondary lymphatic organs to mediate initiation of pathogenic T cell activation (Fig. 2). One interesting and yet unanswered question, which follows from this observation, is whether CCR7 expression is regularly found on subsets of Treg1 and Treg17 cells to facilitate their control of central Th1 and Th17 responses.

## RORγt^+^ Tregs: dynamic players with bifunctional nature

Given the discovery of lineage specific master transcription factors, which define the fate and functional properties of T cell lineages, as Tbet, RORγt, and GATA3, general interest was sparked to comprehensively characterize their expression profile across the various T cell subtypes. In this respect, the surprising discovery was made by Gerard Eberl’s group that a relevant amount of Tregs co-expressed the Treg characteristic transcription factor Foxp3 together with RORγt, one of the defining transcription factors of the Th17 lineage (Lochner et al. 2008). RORγt^+^ Tregs were consistently found by various studies in low, but relevant percentages in the blood of healthy humans (Ayyoub et al. 2009; Voo et al. 2009). Strikingly, they massively expanded during the course of different inflammatory diseases, such as colitis, arthritis, psoriasis, and colonic cancer (Blatner et al. 2012; Bovenschen et al. 2011; Hovhannisyan et al. 2011). In terms of function, RORγt^+^ Tregs were found to produce high amounts of IL10 and potently suppress Teff in vitro. In line with expression of the Th17 characteristic RORγt, however, they also showed the potential to secrete pro-inflammatory IL-17 (Lochner et al. 2008). While these studies suggested to define RORγt^+^ Tregs as a novel Treg subpopulation, their functional relevance in health and disease was not investigated and thus remained speculative.

We thus aimed to close this gap and studied the role of RORγt^+^ Tregs in acute experimental GN. Our analyses in the NTN model revealed a dramatic expansion during the early course of the disease, with RORγt^+^ Tregs comprising up to 50% of the entire renal Treg population. Comprehensive analyses demonstrated that RORγt^+^ Tregs were characterized by a highly activated and immune-regulatory phenotype shown by high expression of ICOS, CTLA-4, CD44, and CD103. However, in line with the previous studies, we also found robust IL-17 secretion by RORγt^+^ Tregs at the site of renal inflammation, as well as in secondary lymphoid tissues (Kluger et al. 2016a). The finding of IL17 production by a Treg subset led to the common hypothesis that RORγt^+^ Tregs resembled a state of trans-differentiation into or from Th17 cells (Komatsu et al. 2013; Valmori et al. 2010; Zhou et al. 2009). However, using adoptive transfer models and fate reporter mice, we could demonstrate that RORγt^+^ Tregs rather constitute a stable and independent Treg subset, which does not derive from nor differentiate into Th17 cells (Kluger et al. 2016a). These findings were fully supported by contemporary studies from other groups (Kim et al. 2017; Ohnmacht et al. 2015; Sefik et al. 2015; Yang et al. 2016). Furthermore, data by us and others uniformly suggest that RORγt^+^ Tregs are induced in the periphery by inflammatory stimuli, such as IL-1ß and IL-6, while they could not be found in the thymus (Hagenstein et al. 2019; Kluger et al. 2016a). Next, we aimed to characterize the functional importance of RORγt^+^ Tregs in GN and performed adoptive transfer studies using the NTN model. Therapeutic injection of RORγt^+^ Tregs into wild type mice led to a significant amelioration of GN, underscoring their immunosuppressive potential despite IL-17 production (Kluger et al. 2016a).

To evaluate the functional relevance of RORγt activation for RORγt^+^ Tregs during crescentic nephritis, we next examined mice with RORγt deficiency solely in Tregs but not in Th17 cells.

Interestingly, Treg-specific inactivation of RORγt led to a marked amelioration, rather than aggravation of GN. In this context, it is, however, important to point out that knocking out RORγt in Tregs, using Foxp3^cre^ x RORγt^flox^ mice, does not deplete RORγt^+^ Tregs, but only leads to their failure to activate RORγt. The Tregs themselves, which are likely to be characterized by activation of further transcription factors, different from RORγt, such as c-Maf, IRF4, and RUNX2/3, remain vital in this setup. Furthermore, a general failure to constitutively activate RORγt, as in Foxp3^cre^ x RORγt^flox^ mice, might result in compensatory changes of the overall Treg transcription profile. Nevertheless, our data indicated some additional pro-inflammatory features of RORγt^+^ Tregs (Kluger et al. 2016a). As potentially underlying mechanism, we found that RORγt-deficient Tregs entirely lost their capacity to produce IL17. In summary, these results revealed the independent and Janus-faced nature of RORγt^+^ Tregs in GN, with both, pro and anti-inflammatory capacities. This prompted us to operationally term them bifunctional Tregs (biTregs). It also needs to be mentioned that unlike knockout of STAT3 in Tregs, silencing RORγt in Tregs did not result in renal or systemic hyper Th17 immunity. RORγt^+^ Tregs thus seem to be different from Th17-specific STAT3-dependent Treg17 cells. This became even more evident by experiments showing that Treg-restricted inactivation of STAT3 had no effect on RORγt^+^ Treg frequency in the NTN model of GN (Kluger et al. 2016a).

Almost in parallel to our observations, two key studies provided further in depth characterization of RORγt^+^ Tregs and identified them as the predominant Treg subset in the colon, which is induced by certain microbiota (Ohnmacht et al. 2015; Sefik et al. 2015). Importantly, specific requirements for their generation and stabilization became evident, which differentiated them from RORγt^+^ Th17 cells and underlined their independent character (Ohnmacht et al. 2015). However, controversial results were reported with respect to biTreg function. Depending on the study and the colitis model analyzed, improved or worsened outcomes were reported for mice with RORγt^+^ deletion in Tregs. Also, in terms of mechanisms used by RORγt^+^ Tregs, many questions and controversies remained. In particular, one study reported an unexpected role of RORγt^+^ Tregs for control of Th2 responses, while the other study did not (Ohnmacht et al. 2015; Sefik et al. 2015).

In order to clarify this aspect and further analyze regulation of immune responses by biTregs, we decided to study the model of chronically developing pristine-induced lupus nephritis. Similar to our results from acute GN, we found significant protection from nephritis in mice lacking RORγt activation in Tregs (Kluger et al. 2017). This again indicated some pro-inflammatory biTreg properties. These might obviously be mediated by their expression of IL-17. However, and in line with the findings by Ohnmacht et al., we also observed strong skewing towards Th2 responses in mice with Treg-restricted RORγt deficiency (Kluger et al. 2017). RORγt^+^ Tregs might thus predominantly suppress Th2 immunity, which is anti-inflammatory in many forms of crescentic GN. It needs to be stressed again though that knockout of RORγt in Tregs does not equal absence of RORγt^+^ Tregs, as explained above. Due to technical limitations, studies analyzing models of true RORγt^+^ Treg deficiency are lacking so far. As a consequence, many questions about the function and origin of endogenous RORγt^+^ Tregs in inflammatory diseases, including GN, thus still remain. Among these are the following: (1) What is the net effect of absence of endogenous biTregs in inflammation? (2) What is the physiological function of biTregs at steady state? (3) Which factors lead to the generation of biTregs? (4) Do biTregs regulate a specific type of immune response? and (5) Why do biTregs express IL-17?

The first two questions have not yet been comprehensively addressed, due to lack of appropriate model systems. Concerning the development of biTregs, the transcription factor c-Maf was recently shown to be essential for their differentiation and maintenance (Neumann et al. 2019; Xu et al. 2018). However, even though biTregs showed high expression of c-Maf, Treg-restricted depletion of c-Maf led to a broader disruption of the Treg compartment, including impaired T follicular regulatory cell (Tfr) differentiation (Neumann et al. 2019). Thus, while c-Maf seems to be important for generation of biTregs, it is not a specific regulator.

In addition to c-Maf, our group could recently demonstrate that classic IL6 signaling was crucial for the generation of biTregs. IL-6-deficient mice, as well as mice with T cell restricted classic IL-6R signaling, showed a profound deficiency to generate RORγt^+^ Tregs under naïve and various inflammatory conditions. Along the same line, in vitro stimulation of Tregs with IL-6-induced biTreg generation and increased their expression of various costimulatory markers. In congruence with these observations, cell type–specific IL-6R deficiency on Tregs resulted in reduced in vitro and in vivo suppressive capacity. Finally, adoptive transfer studies showed virtual absence of biTregs in recipients of IL-6R-deficient Tregs during the NTN model of GN. As a consequence, renal tissue injury was significantly aggravated in these mice (Hagenstein et al. 2019). In summary, biTreg generation is likely to occur in the periphery, rather than the thymus, and depends on IL-6, commensal bacteria, microbial metabolites, and retinoic acid during homeostatic conditions (Abdel-gadir et al. 2019; Al Nabhani et al. 2019; Ohnmacht et al. 2015; Sefik et al. 2015; Song et al. 2020; Yang et al. 2016). Their rapid expansion during inflammatory diseases seems to be particularly promoted by increased availability of cytokines such as IL-1ß and IL-6 (Hagenstein et al. 2019; Lochner et al. 2008). Since the gut microbiome has recently been shown to modulate the course of GN by shaping the renal Th17 response (Krebs et al. 2016), it will be worth studying whether a similar interaction is also found for microbiota and biTregs. A further interesting and unanswered question that remains is whether biTregs suppress a specific type of immune response. We and others could demonstrate that RORγt expression in Tregs was necessary to potently suppress type 2 responses during lupus nephritis and gut inflammation (Kluger et al. 2017; Ohnmacht et al. 2015). More recently, it was additionally postulated that RORγt^+^ induction in Tregs influences intestinal IgA production, potentially in an IL-10 mediated fashion and that RORγt^+^ Tregs might regulate IgA producing plasma cells (Neumann et al. 2019; Ramanan et al. 2020). If these effects, however, can be truly accounted to the function of RORγt^+^ Tregs, or if they just represent secondary effects due to adaptive alterations of the Treg compartment caused by constitutive RORγt deletion in Tregs, will need to be further evaluated. Finally, it remains completely unclear to date, why and with which consequences biTregs secrete IL-17.

**Fig. 3:**
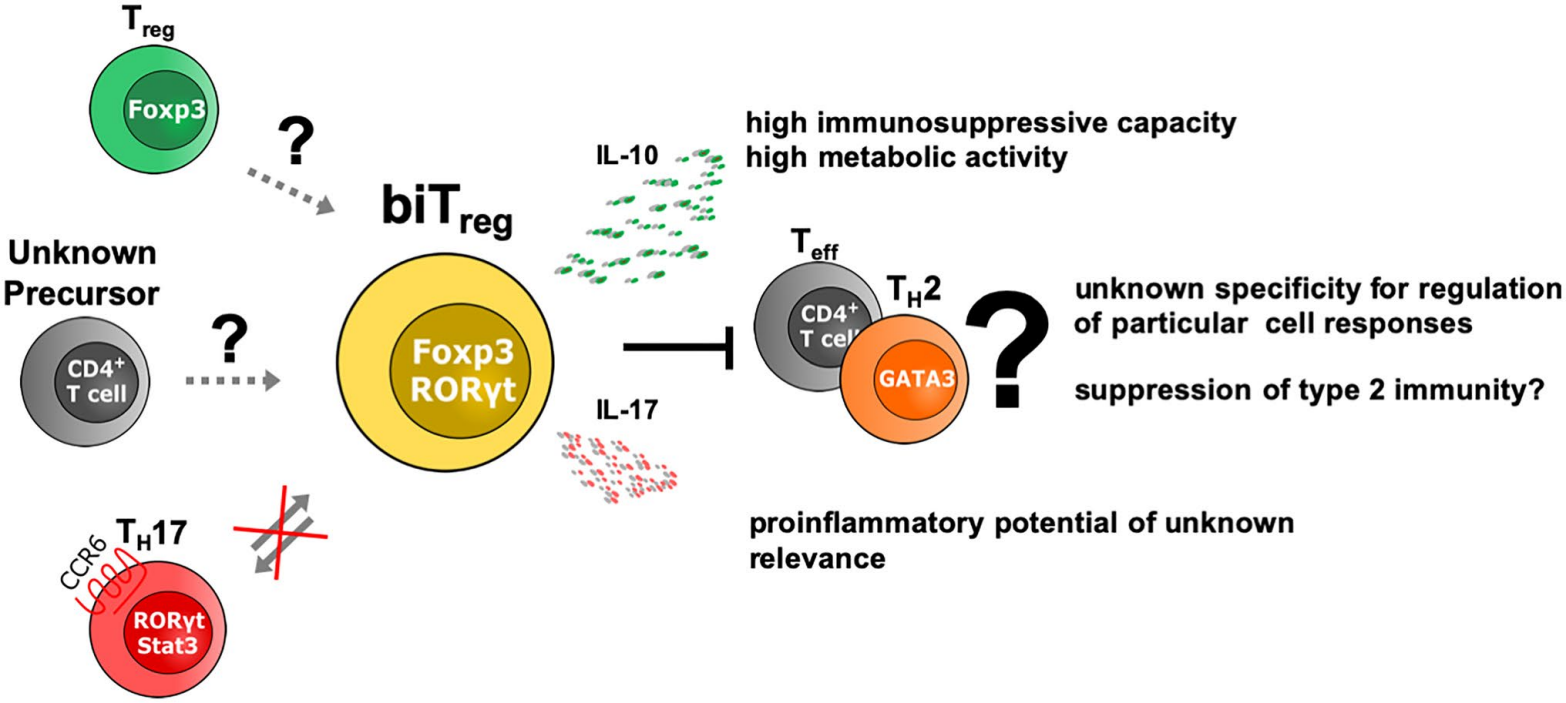
RORγt^+^ Tregs (biTregs) comprise an independent, metabolically highly active effector Treg lineage with enhanced immunosuppressive capacity. Unusual for a Treg, their development depends on IL-6R classic signaling and an additional proinflammatory potential via secretion of IL17 has been reported. However, biTregs do not derive from, or transdifferentiate into Th17 cells. The exact origin of biTregs, as, e.g., development from RORγt^neg^ Tregs or other yet undefined T cell precursors, needs to be explored. In terms of function, predominant suppression of Th2 responses by biTregs has been reported but remains a matter of debate. Specificity for regulation of a distinct T helper cell subtype has not yet been proven

In conclusion, biTregs have been established as independent and unique Treg subtype, which constitutes the largest subpopulation of Tregs infiltrating the kidneys in the early stages of crescentic GN (Kluger et al. 2016a). Furthermore, even though their numbers decline during the later course of inflammation, our data indicate that RORγt^+^ Tregs also play important roles in chronic forms of GN (Kluger et al. 2017). Activation via IL-6 classic signaling was shown to endow them with enhanced suppressive capacity. In addition, however, a robust fraction of biTregs seems to produce potentially pro-inflammatory IL-17 (Fig. 3). The functional relevance of this observation currently remains unknown. Although models mimicking a true biTreg knockout are still lacking, in vitro studies, as well as exogenous transfer approaches suggest an overall protective function. Their early appearance and rapid expansion during GN makes biTregs a promising therapeutic target. However, to facilitate safe biTreg directed therapies, future studies are warranted to further evaluate their potential pro-inflammatory functions. Dampening these, while enhancing their remarkably potent immunosuppressive capacity, might eventually be a successful therapeutic concept, especially for acute and rapidly progressive types of GN.

## Future perspectives

Multiple studies from the recent past have made obvious that Tregs are not a uniform population. Rather, different Treg subtypes with highly differentiated and unique functions exist. However, with respect to GN, it needs to be mentioned that data regarding specific Treg subtypes are mainly based on murine studies, using the experimental models of NTN or pristane induced lupus nephritis. These models certainly show relevant differences in their pathogenesis, compared with their human homologues, ANCA-associated GN, anti-GBM disease, and systemic Lupus erythematosus, respectively. Thus, conclusions have to be drawn with care. Studies functionally evaluating Treg subtypes in other forms of GN, like MPGN, IgA nephropathy, or post-infectious GN, are scarce or absent, due to lack of standardized murine models.

Furthermore, the field is still evolving and many aspects of Treg biology remain unstudied or unknown. Given the discovery of specialized Treg subtypes for control of Th1 and Th17 immunity, one obvious question is whether further specialized Treg populations exist, which are optimized for the control of the remaining T effector cell lineages as, e.g., Th2, Th9, or Th22 cells. Regarding type 2 immunity, the transcription factor GATA3 was shown to be crucial for Th2 cytokine gene expression in CD4 T cells (Zheng and Flavell 1997). Interestingly, however, GATA3 expression in Tregs was demonstrated to have a more profound effect on Treg function and maintenance, rather than regulation of type 2 responses. Treg selective deficiency of GATA3 led to a spontaneous inflammatory disorder and aggravation of colitis, presumably by the inability to maintain Foxp3 expression and thereby resulting in loss of immunosuppressive capacities (Wang et al. 2011; Wohlfert et al. 2011). Instead, IRF4, a transcription factor with broad effects of various key immune cell functions, might be a relevant candidate for Treg-mediated control of type 2 immunity. This was suggested by a study showing that Treg-restricted depletion of IRF4 resulted in overshooting Th2 responses (Zheng et al. 2009). Nonetheless, development and regulation of type 2 immunity seem to be far more complex than initially thought (Bacher et al. 2016). Therefore, it remains an ongoing debate, whether a specific Treg subset for Th2 responses exists. Regarding the renal field, identification of specific cellular players for control of type 2 immunity could be of great importance, since Th2-mediated effects can cause renal fibrosis and progressive loss of kidney function (Liang et al. 2017).

Another interesting question is whether Tregs can directly control activation of myeloid effector cells. In this context, an earlier study suggested that human Tregs were capable of steering macrophage differentiation towards an anti-inflammatory phenotype (Tiemessen et al. 2007). Recent data provided further evidence for this concept and showed that specialized LAG3^+^ Tregs exist in the colon, which contact-dependently restrain pro-inflammatory cytokine production by macrophages (Bauché et al. 2018). Since innate immune cell activation is critical for causing renal damage in glomerulonephritis, a deeper understanding of the underlying mechanisms would help to guide the development of future immunosuppressive agents.

Over the last decades, the increasing knowledge about the potent and diverse immunosuppressive capacities of Tregs opened up the field of Treg-directed therapeutic strategies. Promising results have already been obtained in pioneering human studies (Bluestone et al. 2015; Chandran et al. 2017; Koreth et al. 2011; Von Spee-Mayer et al. 2016). Thus, different approaches to enhance Treg suppression are currently under evaluation. A particularly promising therapeutic option is IL-2 mediated in vivo Treg expansion. A recent study has reported promising results regarding the use of IL-2 for treatment of SLE including Lupus nephritis (He et al. 2020). Further Treg-directed therapeutic approaches include ex vivo expansion and antigen-specific activation of Tregs before reinfusion, ex vivo conversion of Teffs into induced Tregs (iTreg), and genetical engineering of antigen-specific Tregs (CAR-Tregs) (Miyara et al. 2014; Sharabi et al. 2018). In the context of specialized Treg subtypes, it appears particularly promising, to specifically expand Tregs of a desired phenotype (e.g., Treg1, Treg17, biTreg). Further research to identify factors, which initiate Treg subtype differentiation, expansion, and stabilization, is thus much warranted.

## Concluding remarks

Major advances of experimental techniques over the last decades have enabled researchers to better characterize the biology of Tregs in GN (Table 1). It has become evident that Tregs do not constitute a homogeneous population. Rather, different and highly specialized Treg subtypes exist, which are optimized for defined tasks during the course of pathogenic immune responses. Further characterization of the renal Treg landscape in patients suffering from GN will help to advance our knowledge about disease specific Treg differentiation and could potentially reveal additional important subtypes and immunosuppressive mechanisms. The major challenge will be to transfer the findings from model systems to clinical applications and finally develop safe and successful Treg directed therapies for GN.

**Table 1:** Evidence for Treg subtypes in glomerulonephritis

Treg subtype	Disease model	Comment	Reference
Treg17 cells	Crescentic GN (NTN) Lupus nephritis (Pristane)	Stat3 activation leads to CCR6 expression on Tregs in mice and humans and mediates specific control of Th17 immunity	Kluger et al. 2014 Kluger et al. 2016b
Treg1 cells	Crescentic GN (NTN)	Tbet activation leads to CXCR3 expression on Tregs and mediates specific control of Th1 immunity. Tbet enhances Treg fitness	Paust et al. 2016 Nosko et al. 2016
CCR7^+^ Tregs	Crescentic GN (NTN)	CCR7^+^ Tregs enter renal LNs to suppress CCR7 mediated activation of naïve T cells by antigen presenting DCs	Eller et al. 2010
biTregs	Crescentic GN (NTN) Lupus nephritis (Pristane)	biTregs are induced by IL-6 receptor classic signaling and possess high suppressive capacity; RORγt mediates pro-inflammatory potential via IL-17 secretion and suppression of Th2 immunity	Kluger et al. 2016a Kluger et al. 2017 Hagenstein et al. 2019
